# Case Report: Leadless pacemaker implantation in patient with Senning procedure for dextro-transposition of the great arteries

**DOI:** 10.3389/fcvm.2026.1809265

**Published:** 2026-05-28

**Authors:** Hope Kile, Steven Fishberger

**Affiliations:** Department of Pediatric Cardiology, University of Rochester Medical Center, Golisano Children’s Hospital, Rochester, NY, United States

**Keywords:** atrial switch procedure, leadless pacemaker, Micra, Mustard procedure, Senning procedure, TGA, transposition of the great arteries

## Abstract

Patients with dextro-transposition of the great arteries (d-TGA) who have underwent a Mustard or Senning procedure offer unique challenges when pacing needs to be established. This case report describes the Micra AV^TM^ leadless pacemaker placement into the sub pulmonary left ventricle in a patient with d-TGA who had high grade AV block, as well as discusses the benefits and drawbacks of using this device within this population.

## Introduction

Dextro-transposition of the great arteries (d-TGA) is a congenital cardiac malformation consisting of atrioventricular concordance and ventriculoarterial discordance (1). TGA accounts for 5 to 7% of all congenital heart disease. Prior to the development of surgical interventions, 90% of infants born with this defect died within the first year of life. The initial procedures were palliative in nature, including the Blalock-Hanlon Septectomy and subsequently the balloon atrial septostomy. The first successful atrial switch procedure with long term survival, rerouting the pulmonary and systemic venous inflows, was published in 1959 by Ake Senning. This was followed by the Mustard procedure, which used prosthetic material, reported in 1963. However, these procedures resulted in the persistence of a systemic right ventricle and a subpulmonary left ventricle. Attempts at the more physiologic arterial switch operation predated the Senning and Mustard venous procedures, though resulted in 100% mortality until Adib Jatene described the first successful arterial switch operation in 1975. However, the next 5 patients undergoing the arterial switch operation by Jatene did not survive. Therefore, the Senning and the Mustard procedures continued to be the mainstay corrective surgeries for patients with d-TGA for at least another 10 years (2).

One of the most frequent long-term complications in patients who have had an atrial switch operation is bradycardia due to damage to the atrial tissue and sinus node, with at least 15%–20% of patients requiring a pacemaker (3, 4). There are unique challenges with patients who have had a Mustard operation, as approximately 22% have systemic venous baffle stenosis (5), which may be exacerbated by indwelling pacemaker leads. Furthermore, a number of patients who underwent congenital heart surgery, particularly in the early era, experienced significant brain injury. This may have important implications when considering the challenges of cooperating with post operative wound management.

## Case Report

A 38 year old male underwent the Senning procedure for TGA within the first year of life and experienced a neurologic complication, resulting in severe neurocognitive impairment and subsequently is nonverbal. He had no history of syncopal episodes. A Holter monitor in October 2024 revealed persistent sinus bradycardia with marked first degree atrioventricular (AV) block, Mobitz Type I AV block, 2:1 heart block, and one episode of third-degree AV block. The ventricular rate captured ranged from 40 beats per minute (bpm) to 64 bpm, with an average heart rate of 50 bpm. A modified stress test using the Naughton Protocol was performed to examine heart rate response, and demonstrated a blunted heart rate response (resting heart rate was 56 bpm, peak heart rate was 68 bpm, which was 37% of age predicted maximum heart rate) with predominant high grade AV block and occasional PVCs. The stress test was discontinued due to fatigue, however symptoms were unable to be elicited as the patient is nonverbal. An echocardiogram demonstrated patent systemic and pulmonary venous baffles, trivial tricuspid, mitral, and aortic regurgitation, with a dilated and hypertrophied systemic right ventricle with normal systolic function.

Pacemaker implant options were discussed with the patient's mother, given his history of neurocognitive impairment, autism, and nonverbal status with inability to communicate likely symptoms secondary to his high grade heart block. She and the medical team were concerned that if he underwent a surgical procedure with an incision, he would pick at the site, leading to a significant risk of infection and complications. Shared decision was made to implant a leadless pacemaker Micra AV^TM^ Transcatheter Dual Pacing System (Micra TPS, Medtronic, Minneapolis, MN, USA) into the subpulmonary left ventricle.

**Figure 1:**
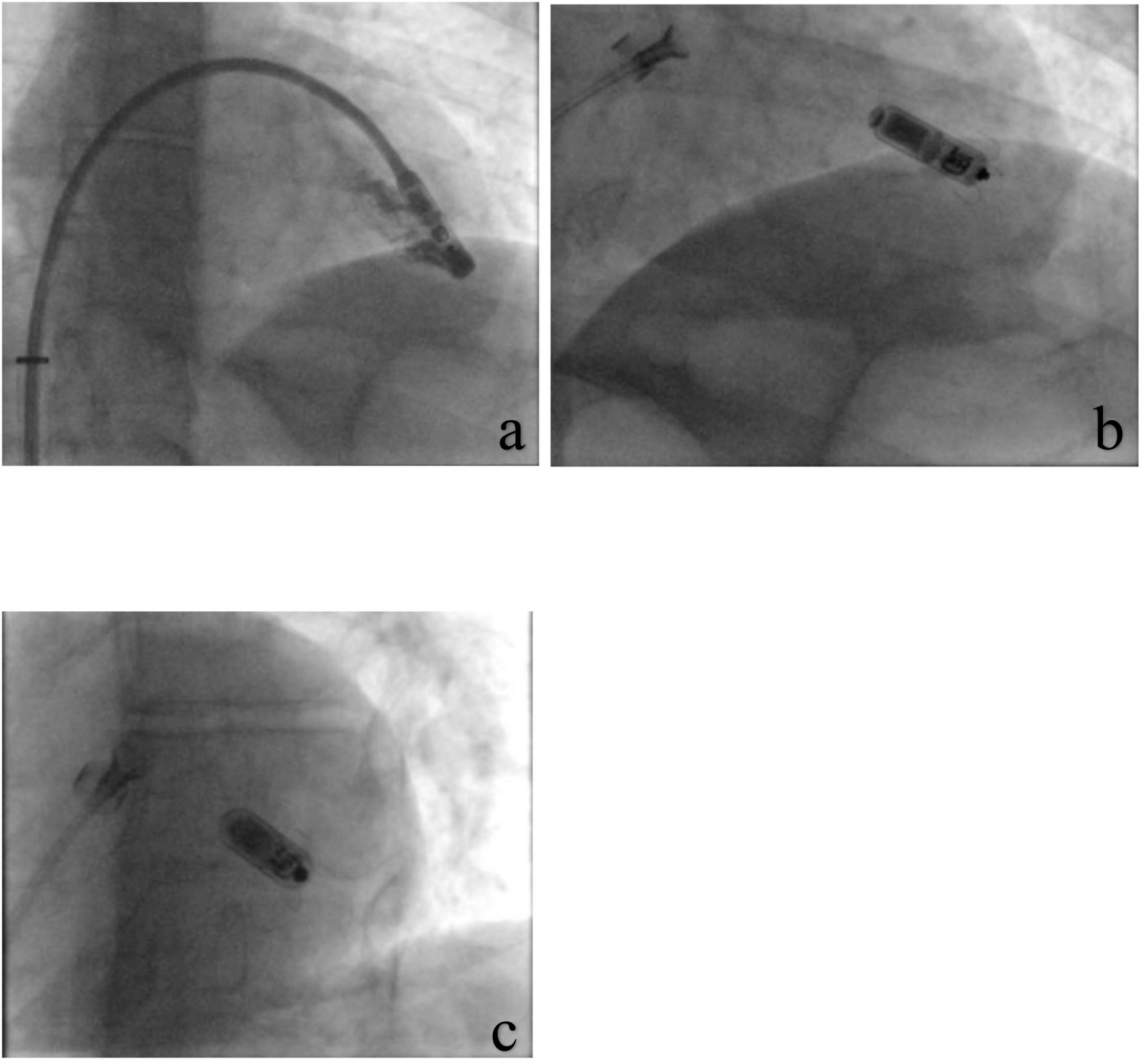
Micra AV^TM^ placement. **(a)** pacemaker system within the LV myocardium with contrast confirmation. **(b,c)** Location of the Micra AV^TM^ delivery device in fluoroscopy during the implantation: **(b)** AP view, **(c)** LAO view.

The patient underwent implantation of the leadless pacemaker Micra AV^TM^ Transcatheter Pacing System under general anesthesia in the catheterization/electrophysiology laboratory. Access was obtained through the right femoral vein under ultrasound guidance, and two Perclose devices were placed as part of the pre-close technique. An Agilis sheath was advanced into the left ventricular (LV) apex, with location confirmed by contrast injection. Two angiographic injections were performed using 15 mL each of iohexol 350 mg/mL, with a total of 30 mL of contrast being utilized. The LV apex was targeted for optimal placement, as the Micra AV^TM^ is a passive fixation device, and the smooth LV septal wall would not provide a trabeculated surface necessary for implantation. An intravenous bolus of 3,000 units of heparin was administered per our institutional protocol prior to implantation of a leadless pacemaker. An Amplatzer superstiff wire was directed into the LV, and the Agilis sheath was removed. Serial dilation of the femoral vein was performed, and a 23Fr delivery sheath was guided to the atrium and the wire was removed. The Micra delivery catheter was directed into the LV apex with contrast confirmation (Figure 1a). The Micra pacemaker was advanced into the myocardium, multiple “tug” tests were performed to ensure that the tines would remain stable within the myocardium, and the system was deployed while retaining connection.

The ventricular sensing, impedance, and pacing threshold were tested, and were >20 mV, 940 ohms, and 0.38 V at 0.24 ms, respectively. The device was tug tested to confirm that the nitinol tines were fixated in the myocardium (Figures 1b,c). The pacemaker was released, and the delivery system was removed. The femoral vein entry site was closed by deploying the two Perclose sutures, achieving hemostasis. The fluoroscopy time was 6.2 min and total dose area product 1,071 *μ*Gym. No complications were observed.

The pacemaker was programmed in the VDD mode with a lower rate limit of 50 beats per minute and upper tracking rate of 105 bpm. He was discharged home the following day after an uneventful overnight observation in the hospital. A remote transmission at 1 month identified atrial undersensing.

The patient was seen for follow up six weeks after device implantation. The capture threshold was 0.38 V at 0.24 ms, pacing impedance 830 ohms, and the ventricular sensing threshold was > 20 mV. Atrial sensing proved unreliable in spite of various programming modifications. The device was programmed in the VVI mode.

Follow up remote transmission several months later demonstrated ventricular sensing 67% of the time, ventricular pacing 33% of the time.

## Discussion

This case report demonstrates that left ventricular implantation of a leadless pacemaker is effective and feasible in patients with TGA following a Senning procedure. This is a valuable option in patients who have neurocognitive delays as there is no incision site and post procedural month-long activity restrictions. Although there is current limited literature regarding placement of a leadless pacemaker in patients with Mustard or Senning procedures, the baffling techniques are similar. The primary difference is the material used (pericardium/atrial tissue versus prosthetic materials), therefore the technique used to place the MicaAV^TM^ in the subpulmonic LV is similar.

To our knowledge, this is only the seventh case report in the literature discussing successful implantation of a leadless pacemaker in a patient with an atrial switch operation (6–8, 17–19), and is only one of two (19) case reports describing Micra AV^TM^ placement in patients with this anatomy to try and achieve AV synchrony. The case reports that demonstrated successful Micra VR^TM^ implantation included a patient post Senning Procedure who had recurrent atrial fibrillation, atrial and tachy and bradyarrhythmias (6), a patient post Mustard procedure with anatomic barriers to transvenous system pacing and symptomatic sinus node disease (7), and a patient post Mustard procedure with superior vena cava obstruction and atrial fibrillation (8). Several other case reports have demonstrated leadless pacemakers using Aveir DR (Abbott) placement and achieving AV synchrony, including in a patient post Senning procedure who had complications with the pocket requiring revision following post transvenous pacemaker system placement (17), and another patient post Senning procedure with symptomatic bradycardia and transvenous lead malfunction (18). The other case report thus far discussing successful implantation of the Micra AV^TM^ was in a patient with a Senning procedure who had complete heart block and pocket infection of his transvenous system, with successful VDD pacing following Micra AV^TM^ placement (19).

Pacemaker implantation in this population may be challenging due to complex intracardiac anatomy and venous access limitations. Following transvenous pacemaker implantation, 30–50% of adult patients develop lead related partial or total venous obstruction, of which 1–3% experience symptoms (9). This may require removal of the system, stent implantation of the veins or superior baffle, and placement of the new device. A leadless pacemaker system avoids this complication.

Infection at a transvenous pacemaker implantation site is a complication that is seen in up to 1% at first implantation, and increases to up to 5% in pacemaker generator replacements (10–14). This can lead to devastating consequences, such as endocarditis and ultimately death. Generator and lead extraction is required, which is especially challenging and high risk in patients who have had an atrial switch operation. A leadless device within the subpulmonary left ventricle can significantly reduce this risk. This is particularly relevant in patients who have cognitive impairment, autism, and/or have the propensity to aggravate a transvenous pacemaker incision site. A leadless pacemaker provides an option with a decreased risk of infectious complications.

The Micra AV^TM^ leadless pacemaker system battery life is expected to be around 8–12 years. Up to three devices per the manufacturer can be placed within the ventricle without any compromise of ventricular function (15), though notably this has been studied only with placement in the right ventricle and not the left ventricle, which may be able to handle less placement of devices but this remains to be seen. If three devices are able to be placed within the left ventricle, our patient could use a leadless pacemaker for the duration of his lifetime, although there likely may be even more breakthroughs within the field, allowing for longer battery life or other options of leadless pacemakers in the future. It is worth mentioning that given the smooth walled structure of the left ventricle comparatively to the more trabeculated right ventricle, placement of a leadless pacemaker with appropriate positioning in patients with an atrial switch operation can be challenging.

The Micra AV^TM^ is the second generation of transcatheter pacing systems, which can provide atrioventricular (AV) synchronous pacing relying on sensing atrial contraction. In order for this second-generation leadless pacemaker to provide this, several novel programming parameters were introduced, including the A3 and A4 window as well as a conduction and activity mode switch. The A3 and A4 window can be manually adjusted based on the patient, where A3 corresponds in timing to the E-wave of mitral inflow on an echocardiogram Doppler and represents passive filling of blood from the atrium to the ventricle, and A4 corresponds in timing to the A-wave of mitral inflow and represents atrial contraction. There have been European guidelines that have been published regarding recommendations for improving AV synchrony in patients who have high degree AV block and intermittent AV block (16), however there are not yet guidelines for congenital heart disease patients, specifically post atrial switch patients. Despite these measures, we were not able to achieve AV synchrony in our patient, and his pacemaker was ultimately set to VVI mode. Likely, the baffles within the atria in combination with high degree AV block presents unique challenges to the atrial sensing capabilities of the Micra AV^TM^, and needs to be investigated further. Unfortunately, ventricular dyssynchrony for our patient could not be addressed using the implanted Micra AV^TM^ device if his pacing burden becomes more significant. If pacing burden does become high along with ventricular systolic dysfunction, we would consider an alternative pacing approach for our patient. VVI mode in a patient with an atrial switch is sub-optimal as they do rely on atrial kick for appropriate hemodynamics, and this is why further device development and consideration of other products such as the Aveir DR (Abbott) need to be considered when discussing implanting leadless pacemakers in d-TGA patients post an atrial switch operation.

Notably, there is a recent case report which presented a patient with d-TGA post Mustard procedure who had a dual chamber leadless pacemaker placed (Aveir DR system, Abbott), where AV synchrony was able to be achieved with the atrial pacer in the left atrial appendage and the ventricular pacer in the left ventricle (17). Despite this being a viable option as well, in younger patients this poses the issue of extracting the atrial leadless pacemaker at the end of its battery life, which has yet to be proven to be possible. Sinus node dysfunction historically has been observed in up to 65% of d-TGA patients following an atrial switch procedure, with more than half of these patients surviving into adulthood and requiring implantation of a pacemaker or ICD (20). With this population growing larger and living longer into adulthood, more research into providing VDD mode in leadless pacemakers is needed.

## Conclusion

In patients with an atrial switch operation with an indication for pacemaker placement, leadless pacemakers appear to provide an alternative to transvenous pacemakers, particularly in patients with neurocognitive delays who may at an increased risk for post-operative complications. This new technology is promising and provides advancement in the field, but still does need further study in this patient population.

## Data Availability

The original contributions presented in the study are included in the article/Supplementary Material, further inquiries can be directed to the corresponding author.
